# The Treatment of Hernia

**Published:** 1893-02-04

**Authors:** 


### PADDINGTON GREEN CHILDREN'S HOSPITAL.

### THE TREATMENT OF HERNIA.

In the preventive treatment of hernia, a little care at the onset of symptoms may save a great amount of time and trouble later on. Screaming and coughing must be checked as far as possible. The bowels ought to be regulated so as to prevent any straining at stool, or excessive distension of the abdomen by gas in the intestine. During an attack of whooping cough, which is one of the most common precursors of hernia, the child should be examined, and any bulging at the usual situations of rupture should be treated by a support bandage and pad. If there is a difficulty in passing water, circumcision or dilatation of the preputial orifice should be done at once. When a congenital hydrocele is present, it must be treated, as the pressure of the fluid keeps open the inguinal canal, and hernia is apt to supervene. Although in hospital practice, the patient usually comes with the hernia fully developed, attention to the above measures forms part of the treatment, the essential principles of which are to support the parts externally, and to diminish, as far as possible the pressure internally. n this paper we shall only refer to some of the more common varieties of hernia.

I. *Umbilical Hernia.*—This form is extremely common in young infants, chiefly in those who are poorly nourished, and in whom, consequently, the natural closure of the umbilical opening has not taken place. There is a special form of truss supplied for this by the instrument makers, consisting of a band of india-rubber which passes round the abdomen, and has an air pad resting on the umbilicus. The ends of the band are fastened at the side by lacing. The variations in size of an infant's abdomen, and the consequent tendency the band has to slip up and down, necessitate for this method of treatment very constant attention so as to maintain continuous pressure. The usual treatment in hospital practice is by means of adhesive plaster on holland. The abdominal wall is thoroughly cleaned, dried, and dusted with starch or zinc powder. A circular piece of block tin, one and a half inches in diameter, is covered with a couple of layers of the plaster, so applied as to completely conceal the sharp edges of the metal, and to leave one surface quite flat with the adhesive side external. A slip of plaster is then cut, two and half inches in breadth, and sufficient in length to extend from the back (posterior axillary line) across the abdomen to the same point on the opposite side. The hernia having been reduced, the pad is applied to the abdomen with the centre of the flat surface resting on the umbilical opening. The long strip of plaster is warmed and fastened to one side of the abdomen, drawn tightly across the umbilical pad to the other side, and after this side has been compressed towards the umbilicus, firmly affixed to it. The effect of this is that whatever changes in size the abdomen may undergo, there will always be a firm pressure over the umbilical pad. With this form of treatment, the child can have his daily bath as usual, care being taken to dry the parts thoroughly around the strapping. Once a week the strapping and pad are changed, and fresh ones applied. In changing the dressing, after the child has had his bath, the strapping is removed by pulling it sharply from the free ends to the umbilicus, and by this means very little discomfort will be experienced. The skin must then be thoroughly dried and powdered as before, and the nurse must see that pressure is maintained all the time over the umbilical opening, so as to prevent the bowel passing out. The treatment must be kept up continuously for from six to eight weeks, by which time the opening will, as a rule, be thoroughly closed; if it is not, the same line of treatment is persevered with. All varieties of truss with a small convex pad which corks up the umbilical opening are condemned, as they simply tend by pressure to make the opening larger, and delay natural healing.

II. *Inguinal Hernia.*—This is treated in the case of children up to three years of age by the wool or flannel truss made and applied at once in the out-patient room, and in older children by a solid truss supplied by the instrument maker. For the former, a skein of Scotch fingering containing from twenty to thirty threads acts very well, but a flannel bandage is more frequently used, as it lasts longer and washes better. The bandage, one inch wide, is folded, and the loop placed over the internal abdominal ring, after the rupture has been reduced; the free ends are then carried up to the anterior superior spine of the same side, round the body one inch below the iliac crests to the other superior spine, through the loop, and then across the perinæum to be fastened behind to the portion that forms the girdle. The bandage when tightened must have the loop resting on the internal ring, and to prevent any tendency to press into the ring, it is advisable to insert at this point a small pad composed of two or three layers of lint below the bandage. A safety pin is passed through the bandage and pad so as to prevent any slipping at the loop. The child can have his daily bath with the truss on, and then a fresh one is substituted, care being taken to keep up pressure at the site of rupture during the process. The wet truss, after being washed and dried, is again ready for use. In cases of double inguinal hernia, two trusses are employed in the manner above described. The mother must in all cases be told that on no account is the truss to be applied with the bowel down. For older children, a solid spring truss, covered with rubber, so that it can be thoroughly washed and dried, is commonly used. This is supplied by the instrument maker, and in ordering it the measurements required are (1) the length from the internal abdominal ring round the back to the opposite ring, in the direction given above in the application of the flannel truss, and (2) the distance between the two internal rings. A double truss is always employed, although the hernia may only be on one side, as otherwise the sound side is apt to give way. The test of a proper truss is that the pressure at the abdominal ring is sufficient to prevent the escape of the bowel, but not so great as to cause atrophy of the parts, with consequent enlargement of the opening. In all cases in children the truss should be worn by night as well as by day. If the hernia is complicated by the presence of a hydrocele, the latter is tapped before the truss is applied. In certain cases no truss will keep up the hernia, and these are treated by the "radical cure." Some of the surgeons perform the radical cure in ordinary cases of inguinal hernia in preference to the truss treatment, as the duration of the latter is often so prolonged that sooner or later, at an unguarded moment, the rupture comes down and the healing process has to commence *de novo*.

III. *Obstructed Inguinal Hernia.*—This is a not uncommon condition in the subjects of ordinary inguinal hernia in whom the bowel has been down for some time, and has got blocked by fæcal accumulation. It can often be relieved by slight taxis after the patient has been put in a hot bath, or after the administration of an anæsthetic. Any manipulation of the parts must, however, be very gently carried out, and if the rupture does not yield readily, the radical cure is usually performed. The details of this operation present considerable differences in the hands of different surgeons, but the general procedure is as follows. An incision is made down to the hernial sac, which is freed from its attachments, and the bowel contained in it is returned to the abdomen. The neck of the sac is then transfixed and ligatured, and the part below is cut off. The neck of the sac, having been pushed well inside the internal ring, is stitched to the abdominal wall, and the stitching of the conjoined tendon to Poupart's ligament completes the closure of the canal. The skin wound is closed in its whole extent by a continuous carbolised-silk suture. The treatment after operation in young children requires great care so as to prevent the urine coming in contact with the wound. A deep dressing of salicylic wool and collodion is applied, which keeps the parts air-tight, and which is intended to remain until the wound is quite healed. Over this is laid a pad of salicylic wool, which is fixed with a spica bandage, and which may be changed if necessary. The nurse is then instructed to watch that no soiled cloths are allowed to remain unchanged for any time, and to report to the house surgeon if the superficial dressing has been stained with urine. After the wound is thoroughly healed, a light bandage is applied for a few weeks. No truss is allowed after the operation, as by pressure it would cause absorption of the new material thrown out, instead of its consolidation into firm fibrous tissue, which is the object of the operation.

